# On the Road to HIV/AIDS Competence in the Household: Building a Health-Enabling Environment for People Living with HIV/AIDS

**DOI:** 10.3390/ijerph120303264

**Published:** 2015-03-18

**Authors:** Caroline Masquillier, Edwin Wouters, Dimitri Mortelmans, Brian van Wyk

**Affiliations:** 1 Research Centre for Longitudinal and Life Course Studies (CELLO), University of Antwerp, Sint-Jacobstraat 2, Antwerp 2000, Belgium; E-Mails: edwin.wouters@uantwerpen.be (E.W.); dimitri.mortelmans@uantwerpen.be (D.M.); 2 Centre for Health Systems Research and Development, University of the Free State, 205 Nelson Mandela Drive, Park West, Bloemfontein 9300, South Africa; 3 School of Public Health, University of the Western Cape, Robert Sobukwe Road, Bellville 7535, South Africa; E-Mail: bvanwyk@uwc.ac.za

**Keywords:** HIV/AIDS, HIV/AIDS competence, household support, health-enabling environment, HIV/AIDS competent household, care continuum, treatment adherence, South Africa

## Abstract

When aiming to provide chronic disease care within the context of human resource shortages, we should not only consider the responsibility of the individual person living with HIV/AIDS (PLWHA) but also the capacity of the social environment to actively encourage a lifestyle that fosters health. In this social environment, extensive efforts are thus required to increase HIV/AIDS knowledge, reduce stigma, stimulate HIV testing, improve health care-seeking behavior, and encourage safe sexual practices—described in the literature as the need for AIDS competence. In accordance with socio-ecological theory, one cannot restrict the research focus to communities, as AIDS competence studies should also incorporate the intermediate household level. In responding to this research need, the aim of this article is to conceptualize an “HIV/AIDS competent household” based on qualitative interviews and focus group discussions conducted in a township on the outskirts of Cape Town, South Africa. Our results show that a household’s supportive response to disclosure allows a patient to live openly as HIV positive in the household concerned. This may mark the start of the road to HIV/AIDS competence in the household, meaning the PLWHA receives sustainable support throughout the care continuum and positive living becomes the norm for the PLWHA and his or her household. A feedback loop might also be created in which other household members are encouraged to be tested and to disclose their status, which is an important step towards a sustainable response to HIV/AIDS-related challenges. Despite the fact that this road to HIV/AIDS competence at the household level is fragile and prone to various barriers, this article shows that the household has the potential to be a health-enabling environment for PLWHA.

## 1. Introduction

Since the first cases of AIDS were described there has been much to celebrate with regard to the progress made in the treatment and prevention of the disease [1]. Roll-out of antiretroviral treatment (ART) in many countries offers the promise of normal life expectancy for people living with HIV/AIDS (PLWHA) who successfully navigate the care continuum [2]. This care continuum determines the trajectory a patient will take after an HIV-positive test [2,3]. Scholars have identified four essential steps along this continuum: (1) linkage from testing to enrollment in care, (2) determination of ART eligibility, (3) ART initiation and (4) adherence to medications to achieve viral suppression [3]. In order to optimize health outcomes for the patient and to prevent transmission to others, each step of the care continuum must be completed. 

However, it has been noted that many patients are lost from the continuum at each stage, so that few actually achieve undetectable viral loads [4]. Data available from various programs are insufficient to accurately characterize the continuum of care [4]. In relation to an abbreviated care continuum, data show that of all PLWHA in sub-Saharan Africa aged 15 years or older, 45% know their status, 39% receive ART and only less than one out of three (29%) have suppressed their viral load [5]. Attrition of patients across the HIV care continuum severely undermines the overall effectiveness of HIV programs [3,6]. Multiple barriers have been found at various levels which explain the failure to achieve the entire continuum [3,6]. To be successfully treated, sufficient attention to the psychosocial dimensions of chronic disease care is required [7,8,9]. These encompassing care needs, combined with a high prevalence of HIV/AIDS, place increasing pressure on an already stretched health care system. 

When aiming to provide chronic disease care within the climate of human resource shortages, we should not only consider the responsibility of the individual, but also their social environment and its capacity to actively stimulate a lifestyle that fosters health. In this social environment, extensive efforts are thus required to increase HIV/AIDS knowledge, reduce stigma, stimulate HIV testing, improve health care-seeking behavior, and encourage safe sexual practices—described in the literature as the need for *AIDS competence* [10]. AIDS competence reflects the idea that “the likelihood that people will choose health-enhancing practices depends not only on individual-level factors, but also on the extent to which they live in social environments that enable and support this choice” ([11], p. 10). Achieving AIDS competence cannot be done by individuals alone, it is a group phenomenon [10]. In the words of Weihs *et al*.: “health decisions are not made in social isolation” ([12], p. 16). Adaptation to HIV/AIDS is the outcome of continuous interactions between the individual and his/her immediate environment [13]. These ideas are rooted in a socio-ecological perspective, which emphasizes the interrelatedness and interdependency of individuals and their social environment, which in turn affect disease management and its outcomes [12,13,14,15]. 

In this regard, many studies have focused on enabling communities to make the right behavioral choices as an intrinsic component of a durable and sustainable HIV/AIDS strategy [16,17,18]. An AIDS competent community provides a context—which is characterized by a sense of within-community solidarity [19]—in which local people work together to face the challenges of HIV/AIDS [20] by: recognizing the reality of HIV and AIDS; building capacity to respond to HIV and AIDS; exchanging and sharing knowledge and skills; reducing vulnerability and risks; and living to their full potential [18]. Lamboray and Skevington argue that “the more AIDS competent a community becomes, the more likely they will be to have a range of good outcomes” ([10], p. 519). Emerging evidence shows that the development of AIDS competent communities yields various positive outcomes, ranging from less HIV infection and more care and support for people living with or affected by HIV/AIDS to improved quality of life in the communities affected [21]. 

However, with a socio-ecological perspective in mind, one cannot restrict the research focus to communities if we are to study HIV and AIDS competence comprehensively. As patients seldom live in isolation from the household—which affects different aspects of disease management—not only communities but also households should be taken into account when building social contexts that enable and support the choice of health-enhancing practices [12,13]. Perhaps more than any other epidemic, HIV is an illness that affects the whole household [22]. The impact of an individual being infected with HIV radiates across the entire household system. The direct and indirect impact of transmission risk, care burden, social stigma, physical illness and emotional distress is shouldered by the various household members [22,23,24,25]. In the words of Bor *et al.*, “HIV infects individuals and simultaneously affects a whole network of significant relationships” ([26], p. 168).

According to AIDS 2031 Social Drivers Working Group “no longer will we be able to address AIDS in a short-term emergency mode. Future endeavors will need to appreciate that AIDS epidemics are “long-wave” events that evolve over generations” ([11], p. 16). Thus, it is indispensable for sustainable long-term success that PLWHA live in households that support and enable the choice of health-enhancing practices, *i.e.*, HIV and AIDS competence. To the best of our knowledge, ours is the first study to conceptualize an HIV/AIDS competent household in response to the above-mentioned research need.

## 2. Key Concepts

### 2.1. AIDS Competent Community 

An AIDS competent community is defined by Campbell *et al*. as “a social setting in which people are most likely to work collaboratively to optimize HIV/AIDS prevention, care and treatment” ([27], p. 124). Local networks, norms, and relationships between the sexes and among generations are assets that even the most resource-poor settings can exploit in order to optimize communities’ use of prevention, care and treatment services [27,28]. However, while developing community AIDS competence has the potential to create a health-enabling environment [21,27], research has demonstrated that it is “a long-term and delicate process” [28] subject to several HIV-specific barriers (e.g., HIV-related stigma and discrimination and local HIV-related myths [2,6,20]) and non-HIV-specific challenges (e.g., community tensions [29] and a lack of resources and skills [30]). 

Following a community’s recognition of HIV, which might be the result of collective shock over the loss of community members [10,18,21,31], change agents or agencies such as NGOs and PLWHA themselves need to raise the community’s awareness of their capacity to deal with the problem using awareness-raising techniques designed by local people for their own use [10]. After raising awareness, community members should then act to change attitudes and behavior [10] in order to mitigate the impact of HIV and reduce vulnerability to further HIV infections [21]. Campbell *et al*. identified five key features of an AIDS competent community that underpin these behavior changes: (1) gaining, sharing and translating knowledge about HIV/AIDS into health-enhancing behavior change [11,18,19,32]; (2) creating a social space for dialogue and critical thinking [19]; (3) fostering a sense of ownership of the problem and responsibility for contributing to its management; (4) building solidarity and a common purpose [11,19,20,32]; and (5) forming partnerships with bridging social capital [10,11,19,32]. 

In social settings characterized by a sense of within-community solidarity, HIV/AIDS prevention, care and treatment can be optimized [11,19,27]. Within such communities, stigmatization is challenged [19]; PLWHA are also less likely to respond to the epidemic with fear and denial and more likely to feel confident enough to seek out information about prevention and/or testing [19]. As vulnerability to HIV decreases and behaviors change, the incidence of new infections declines [21], as does the number of new AIDS cases and other infections such a sexually transmitted diseases and tuberculosis [10]. In order to sustain AIDS competence over time, responses from the community must change as the nature of the epidemic changes within the community [21]. This involves an intrinsic feedback loop in which community action encourages a subsequent cycle of action, gradually generating an AIDS competent society [10].

### 2.2. Households: Challenged, not Damaged

Due to the roll-out of ART, HIV/AIDS is no longer defined as an acute fatal disease but as a chronic illness. Managing chronic conditions is increasingly seen to be the responsibility of the individual and the household in which they live, who must be encouraged to actively engage in a lifestyle that fosters health [33,34]. This increasing chronic status of HIV has shifted priorities in the household “from planning for inevitable and relatively imminent death to construction of a life encompassing maximal function and well-being” ([24], p. 69). Despite the crucial role played by the household social context, few studies have investigated the level of the household in HIV/AIDS disease management [13]. 

In the conceptualization of AIDS competent communities, attention has shifted from community deficits to strengths [13]. This study will shift in a similar way to the strengths of a household, viewing households “as challenged, not as damaged” ([35], p. 22). In this study, households are conceptualized as being resourceful in providing care and capable of addressing the challenges they face [36] by developing and deploying their own strategies [37]. In line with Niehof, we rely on Rudie’s definition of a household as a “co-residential unit, usually family-based in some way, which takes care of resource management and primary needs of its members” (Rudie in [38], p. 490). The two key household attributes used in our study are spatial proximity and day-to-day interaction, since these characteristics were found to be vital to a household’s ability to fulfil the primary needs of its members, such as care, on a daily basis in the context of HIV/AIDS [38,39].

Like communities [27], households may have assets that assist in the building of AIDS competence. In responding to the challenges of HIV/AIDS, previous research has shown that households have immense potential to provide strength and support [40]. One of these strengths—even in the most resource-poor settings—is bridging and bonding social capital [39,41]. Bridging social capital has been found to provide “access to new information and resources, enhancing people’s actual control and improving their ability to solve various problems” ([42], p. 122). Bonding social capital is considered important in the provision of social support and in mobilizing solidarity [43]. A supportive household environment has been shown to motivate ART adherence [44] and to play an important role in supplying messages of hope [45]. However, like communities [46], households may vary in their capacity or readiness for collective action. In addition to an immense potential for strength and support during times of need and crisis [40], the existing literature has indicated that such social networks can also be a potential source of stress [47] and stigma [48]. In other words, interaction within the household can be detrimental as well as helpful [49]. 

## 3. Methods

### 3.1.Ethical Approval

Ethical approval was granted by the Ethics Committee of the University of the Western Cape (13/10/55). Before enrollment in the study, the informed written consent of all participants involved was obtained. Information about the study, its design and aspects such as voluntariness and confidentiality were distributed by means of an information leaflet, available in both English and the local language and explained in an understandable way to the respondents. Respondents who completed the interview or the focus group discussion received a voucher as a token of appreciation for their time and collaboration. To prevent the risk of inadvertently disclosing study participants’ HIV status and to make the respondent feel as comfortable as possible, the respondent could choose the time and place for the interview. Interviews were completed in English, the local language or a mix of both languages, depending on the preference of the respondent. 

### 3.2.Context and Setting

This study is part of a larger project that focused on the interaction between a patient’s household environment and the treatment adherence support provided by community health workers (CHWs) who were employed by a large non-governmental organization (NGO). To be eligible for enrollment in the study, patients were required to meet the following selection criteria: being 18 years of age or older; being HIV-positive; and participating in the NGO’s treatment adherence support program. 

The NGO operates in three different areas of the Cape Metropole. The HIV prevalence in this health sub-district of the Western Cape Provincial Department of Health is 19.1% [50]. The study site (Klipfontein/Mitchell’s plain) was purposely selected, aiming to cover all the health facilities providing TB care and ART, to which the CHWs studied are linked. These health care facilities, operated by the Provincial Department of Health were primarily nurse-driven—reflecting task-shifting as one answer to the national shortage of health care workers. The majority of people living in this informal settlement had no formal street address, while some formal houses present in this impoverished area on the outskirts of Cape Town were also included.

### 3.3. Data Collection

A combination of interviews and focus group discussions was used in this study to achieve data triangulation. The findings of the various qualitative research methods allows us to look at the same topic from different angles, rendering the results more valid [51]. 

To start, 13 CHWs were followed on their daily tasks of visiting patients to provide treatment adherence support. Of the 73 houses visited, 48 patients or their treatment buddies were home to attend the community-based adherence support session. To give patients the time to reflect on the decision to participate in the interview, and to make the respondent as comfortable as possible, the respondent chose the time and place for the interview. Of the 48 persons observed during the community-based adherence support session, 41 agreed to participate in an interview on a subsequent day. Nine of these interviews did not go ahead, however: three patients declined to participate further in the study, four did not attend on the day of the interview and could not be tracked, and two had a job and were unable to attend the interview. As a result, a total of 32 in-depth interviews were conducted with patients living with HIV/AIDS, using an interview guide adapted on the basis of lessons learned from pilot interviews and pilot observations.

Interviews were semi-structured to ensure that the same topics were covered in each, while allowing unanticipated material to emerge. Semi-structured qualitative interviews with the patients ranged from half an hour to one and a half hours. After obtaining informed written consent from the participants, all but one interview was audio taped. Respondents completed a short interviewer-administered survey to provide basic socio-demographic information before participating in the semi-structured qualitative interview. The domains explored through the qualitative interview included HIV testing, disclosure, household involvement and treatment adherence support. Some of the questions in the semi-structured interview related to personal and sensitive issues. The respondent was free to decline to answer any specific question if he or she felt that the information was too sensitive or personal. Furthermore, the principal interviewer and the male and female translators paid specific attention to this aspect and remained sensitive to the limits of the participants. 

In addition to the in-depth interviews, four focus group discussions were held with 36 out of the 39 CHWs working for the NGO at the four health facilities in the study area. The focus groups discussed topics that emerged during the interviews, such as engaging in the treatment adherence sessions and the advantages and disadvantages for the patients, and the CHW’s experiences of the social environment of the patient. 

### 3.4. Respondents

The majority of the patients interviewed were female (23 out of 32 respondents). When assessing the highest level of education achieved, the majority of the patients had enjoyed some or completed secondary education. On average, the respondents were 35.6 years old, ranging from 21 to 59. All patients were black and spoke English and/or a local language. 

On average, households had four members. Twelve respondents were not in a relationship, while 11 PLWHA in this sample were living with their partner, and nine were in a relationship but not living with their partner. Except for two patients, none of the respondents had a paid job. Five PLWHA were receiving a disability grant, while nine were waiting on the response to their application for this grant.

Ten patients had previously defaulted their ART, of which one was still not following treatment at the time of the interview. Treatment duration ranged from less than a month to more than six years on ART. Most patients were receiving the fixed dose combination. Seventeen patients reported they had side effects from the treatment, and 13 respondents were on both ART and TB treatment. All patients received visits from a CHW to help with adherence.

### 3.5. Data analysis

The audio recordings allowed us to produce a detailed transcript of each interview—assuring an accurate understanding of what was said—which was the basis for data analysis. The recordings of the interviews and focus group discussions were transcribed verbatim and when necessary translated into English. A sample of translations was back-translated to the local language for a quality check. Transcripts, moderators and observation notes were imported into NVivo, version 10, for analysis. Data collection and data analysis were alternated to inform the subsequent interviews and focus group discussions and to assess when data saturation was reached.

The aim was to propose a middle-range theory which was context specific and applicable to the population studied. Based on the literature and qualitative data analysis, we will outline the conceptualization of a health-enabling household for patients living with HIV/AIDS below. More specifically, we will focus on those households in which at least one member is living with HIV/AIDS. Non-co-residing partners, who stayed irregularly in the house of the patient, were also taken into account.

The data was analyzed carefully by reading and rereading the transcripts of the interviews and focus group discussions in accordance with the Grounded Theory procedures described by Strauss and Cobin [52]. First, the data was open coded. In this phase of data analysis, primary information categories which remain close to the original data were constructed. Codes of a sample of transcripts were compared with another researcher’s codes and similarities and differences discussed. These open codes were then categorized in the axial coding phase to identify patterns and regularities emerging from the data. The categories which emerged from the axial coding were integrated in the subsequent phase of selective coding. Concepts were systematically refined as the data were collected and analyzed. In this process, specific attention was paid to remaining close to the gathered data. The findings were consolidated to account for meaning in the patterns, in the light of existing scientific literature on AIDS competent communities. In this regard, sensitizing concepts were used, which indicated the paths to follow without allowing these concepts to dominate or steer the analysis [53]. Coding and analysis were performed concurrently with the development of the figure illustrating the results. The final analytical figure can be found in the results section.

## 4. Results

**Figure 1:**
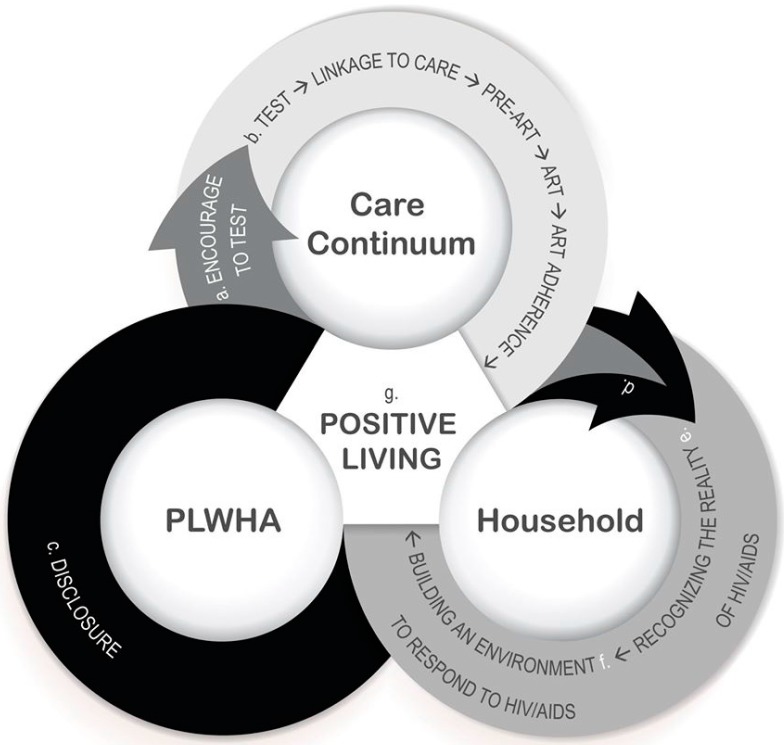
On the road to HIV/AIDS competence in the household.

An HIV infection not only affects the patient but also other members of the household. By its very nature, HIV is associated with a number of characteristics which affect intimate relationships. Infection represents a risk to the entire household and accordingly there is a need to prevent the spread to a partner or children. However, respondents did not see their household as being solely dominated by the illness. They indicated the importance of the household’s involvement in their life with HIV/AIDS. Below, we will outline the road to HIV/AIDS competence in the household, which is illustrated in Figure 1.

“I feel like I can defeat it now, I am more positive. […] Because of the support that I have been getting here in the household.”

*(Male PLWHA, 24)*

### 4.1. Context 

The readiness for HIV/AIDS competence is dependent on a myriad of interwoven factors. The results reveal both HIV and non-HIV-related dynamics in the households, which provide a breeding ground for HIV/AIDS competence. First, pre-existing household dynamics, such as emotional connectedness, honesty, supportive relationships, good internal functioning, a climate of trust and open communication, can all be helpful in the management of HIV/AIDS. Second, in addition to these non-specific HIV characteristics, pre-existing knowledge of HIV/AIDS was also an important factor contributing to the development of HIV/AIDS competence. Moreover, HIV-related precedents, such as other people living with HIV/AIDS in the household of the respondent, contribute to the maturation of HIV/AIDS competence in the household. As a result of the loss of a household member or another HIV-positive household member previously disclosing their HIV-positive status, an awareness of HIV/AIDS in the household may already exist. In a household which has already been through the process of building up HIV/AIDS competence, the way is already paved for the new HIV-positive household member. Nevertheless, the disclosure by a newly identified HIV-positive person to fellow household members is still the first, difficult step required, as we will see below:

“In some instances, you will find that in other families there are more than three or four people taking the same pills and then it’s easy, they always support each other. And that there is not stigma because everyone in the house is just open about it.”

*(Community Health Worker, Health Facility 3)*

### 4.2. Process 

The development of an HIV/AIDS competent household is an ongoing interactive process, in which the various steps set out below may be taken and retaken. How these intrinsic feedback loops develop is specific to each particular household in the sample. HIV/AIDS competence is built in the interaction between the PLWHA and his or her household. The road to AIDS competency begins with the recognition of the reality of HIV and AIDS by the household, as illustrated in Figure 1. Without such recognition, one cannot create an environment that is able to respond to HIV/AIDS and results in a positive lifestyle for the household members. 

A household that is positively involved at the start of the care continuum (**b**), for example by encouraging a household member to be tested (**a**), provides a good environment in which to build up HIV/AIDS competence. A household’s supportive response (**d**) to disclosure (**c**) allows a patient to live openly as HIV positive on ART in the household concerned. This may mark the start of the road to HIV/AIDS competence in the household (**e**), meaning the household constitutes a health-enabling environment (**f**) which provides sustainable support to the patient throughout the care continuum (**b**) and positive living becomes the norm for the PLWHA and his or her household (**g**). A feedback loop might also be created in which other household members are encouraged to be tested (**a**) and to disclose their status (**c**), which is an important step towards a sustainable response to HIV/AIDS-related challenges. How these feedback loops develop is specific to each particular household and depends on the household context and other factors.

#### 4.2.1. Recognizing the Reality of HIV/AIDS in the Household

A key step on the road to HIV/AIDS competence is transforming the individual’s HIV status into a shared reality in the household. A household must first acknowledge the existence of the disease in its midst. In this process, others become involved and thus stigmatization becomes a threat. If there is a need for stigma management, a patient may attempt to regulate his or her roles in the social environment by adopting a hybrid identity. In other words, the PLWHA will attempt to hide their HIV status when fearing stigmatization, but embrace the identity of an HIV patient on ART when feeling safe at home. Thus, the patient uses disclosure as a means of controlling his or her hybrid identity. Adopting such a hybrid identity manifests itself in different ways among the respondents. Some patients only disclose to certain people in the household and attempt to hide their condition from the other household members. Other PLWHA keep their positive status a private household concern embracing the identity of an HIV patient only in the safe environment of the house. In the words of one respondent:

“I am living with AIDS in my house, not in their house.”

*(Male PLWHA, 24)*

However, markers of HIV/AIDS, such as visible signs of illness, the presence of ART tablets, a clinic card, or visits by a CHW, render this control over their hybrid identity more difficult, as the patients in this study demonstrated. Clearly, HIV/AIDS-related markers can stimulate or force disclosure, as they trigger questions, or because the respondent anticipates the concerns articulated by household members:

“I decided to tell her [partner] because she asked me lots of questions about my pills because I separate other pills from ARVs. I told her she must stop interfering to my things. But the other day we were both happy and I’ve decided to tell her the truth that I’m on treatment and I am using ARVs.”

*(Male PLWHA, 52)*

The process of disclosure is distinct for each patient. While in this sample an open personality and the values of the patient were a facilitator of disclosure, the decision to disclose also involved careful consideration of its potential negative consequences weighed against its potential advantages. On the one hand, disclosure brings a multitude of possible stressors, from fear of stigma, gossip and discrimination to disruption of relationships. Disclosing one’s positive status can provoke questions of sexuality or blame and the associated fear of rejection. Fear of rejection is especially the case when women are economically dependent on their partner. Thus, these perceived disadvantages are taken into account when deciding on disclosure. PLWHA in this sample who were aware of their source of infection and were infected as a result of unprotected nursing an HIV-positive patient were more open about their status than those who contracted HIV through sexual intercourse. On the other hand, the perceived advantages that led respondents to disclose their status concerned encouraging their significant others to get tested, as well as a way to open up the possibility of accessing support, ranging from emotional support and care to financial and material support provided by household members:

“I talk to my cousin because we are very close and I trust him a lot. Whatever I share with him, he does not spread any rumors about me or discuss it with his friends. […] My cousins are two but I share my problems with the younger one Sipho and the other one is an alcoholic, so I don’t like to talk to the older one.”

*(Female PLWHA, 27)*

This evaluation of the consequences they expected to face when disclosing their HIV-positive status was based on the pre-existing household context in which the respondent lived. PLWHA in the study tend to first disclose to the person to whom they talk to in general when feeling sad or distressed and with whom they have a positive pre-existing relationship. Furthermore, HIV-related knowledge in the household and HIV-related positive precedents—for example, if the household had responded positively to disclosure by another household member, or if another person in the house was on ART—created a stimulating climate that encouraged the sharing of one’s status. Positive pre-existing household dynamics also facilitated disclosure. Not only because the patients in the sample felt more comfortable, but also because other household members had noticed relatively quickly that the PLWHA was behaving differently, as this respondent testifies:

“I trust him [cousin] so much, even that day I was from the clinic he noticed that I was not well. He always comes home earlier than the other one [cousin] and he asked what was bothering me. I lied and say no I have a headache and he says whatever it is, I must tell him because I am his family, he deserves to know. And I told him.”

*(Female PLWHA, 27)*

While disclosure is a prerequisite, it is not sufficient in itself to start building HIV/AIDS competence. The initial response to disclosure can evolve over time, from disbelief or a negative reaction to a more supportive response. A supportive response by household members is required if HIV/AIDS competence is to be built in the household. The supportive responses to disclosure in this sample range from a neutral acknowledgment of the reality of HIV/AIDS to a sad or shocked response which included, at the same time, the promise of support. As a consequence of the creation of a hybrid identity, self-selection protected most of the patients in the sample from experiencing stigmatizing responses to disclosure:

“There is no one [judging], because there is no one from outside who knows my status. It’s only my family that knows.”

*(Female PLWHA, 22)*

#### 4.2.2. Change Agents

When there is a need for stigma management, it is important for the respondent to preserve their desired hybrid identity. Aiming to regulate the involvement of the household in disease management, the PLWHA often acts as a *gatekeeper*. In this regard, in the majority of the households in the sample the PLWHA was the change agent who created awareness and openness about the disease and the need for behavior change to prevent further transmission to others in the household.

However, in some households in the study another household member was the motor that started the move towards HIV/AIDS competence. A household that is positively involved from the start of the care continuum provides a good climate to build HIV/AIDS competence. A patient who is triggered directly or indirectly by household members to have a test will disclose his or her status more readily to these household members. A household member that is already known to be living with HIV/AIDS can be an indirect trigger for a test. Based on the knowledge about HIV/AIDS of a household member, the encouragement to have a test may also be much more direct, as this patient testifies:

“My mother has noticed that I lost weight and I had a skin rash; she forced me to go to the clinic and I didn’t want to go. One day she took me to the clinic and she was watching me like a hawk because she thought I was going to run away. The nurse attended me and she asked me if I was sick and I said I was suffering from a stomach ache. The nurse asked me if I was willing to do the HIV test and I agreed on that.”

*(Female PLWHA, 21)*

#### 4.2.3. Building a Household Environment to Respond to HIV/AIDS 

Awareness and acknowledgment of HIV/AIDS in the household through a supportive response to disclosure are prerequisites for building an HIV/AIDS competent household. HIV/AIDS competence arises from these changes to behavior that are sensitive to household dynamics and are based on the following five features of an AIDS competent household - in line with the characteristics outlined at the community level by Campbell *et al.* [19,27,32]: bridging social capital; exchange and sharing of knowledge and prevention skills; ownership and responsibility; social space for dialogue and critical thinking; and solidarity and common purpose.

#### 4.2.4. Bridging Social Capital

Bridging social capital is needed to access resources from outside—such as other households, local NGOs working on HIV prevention and AIDS care, and health care organizations—that can support the household in its effort to support the patient. Bridging social capital is required to have the HIV test itself. The majority of the patients in this study sought care after they had developed the symptoms associated with a low CD4 cell count, started feeling sick, or had another health complaint, such as tuberculosis (TB) or a Sexually Transmitted Infection (STI). Pregnancy or family planning led women in the sample to a health facility, where the test was initiated by the health care provider. Such provider-initiated testing led more women to be tested earlier than their male partner in this study. The respondents also received more accurate information on the infection and the response to it from bridging partners such as nurses, doctors, community health workers, fellow patients in the waiting room, and TV and radio programs. 

Bridging social capital is also required to access treatment and condoms, for example. Respondents in this study received free ART, as provided in the public health care program. Recently, the pill burden was reduced for new patients who receive the fixed-dose combination. All respondents also received treatment adherence support visits from CHW at their home. In the counseling sessions preceding the start of ART, the patients learn more about HIV/AIDS and ART, and attitudes and practices such as prevention. Inviting a treatment buddy, chosen by the patient, to the counseling sessions may encourage the household to engage in the disease management. A treatment buddy is usually someone with close personal ties to the patient, who is aware of the patient’s status and will provide support once ART begins. This is the only moment that the involvement of the social environment of the patient is formerly built into the care continuum. Some patients’ household members took part in the clinic visits from the start, while for others this actually led them to disclose their status:

“I only told her [sister] because the clinic asked me to come with her, because she had to sign for me.”

*(Male PLWHA, 52)*

#### 4.2.5. Exchange and Sharing of Knowledge and Prevention Skills

Awareness of HIV/AIDS goes hand in hand with knowledge of the disease being shared by household members. In this regard, in a climate of open communication the respondents will disseminate the knowledge they have gained about HIV/AIDS and prevention skills from bridging social capital to other household members. Moreover, some of the PLWHA in this study also received advice from fellow household members; in particular, from another person living openly with a positive status in the household:

“I told my wife about it. The counseling was done and they told me about the same thing that she told me, that actually was not new to me.”

*(Male PLWHA, 46)*

The sharing of HIV/AIDS-related knowledge by household members about the infection, prevention, treatment and its side effects is required to build an environment that is responsive to HIV and AIDS. An increase in HIV/AIDS-related knowledge supports the gradual process of normalization of HIV/AIDS in the household. An understanding of the nature of the disease by the patient and fellow household members is important for demystifying the disease, eradicating stigma and engaging in successful strategies that start or sustain preventive behavior in that context. Given high co-infection rates, respondents who had personal experience of TB also had knowledge about TB symptoms and treatment, which may be important to improve the case detection rates. Some patients in the study started acting as *household health advisors*. Women are more likely to act as a household health advisor to their partner or children than are men in this study. In addition to sharing information on HIV/AIDS, patients in the study encouraged other household members to get tested, prevent the spread of HIV by distributing and using condoms, wear gloves when caring for HIV-positive patients and to take ART when positive. Below, one female patient talks about a conversation with her children:

“I tell them [children] to use condoms. I want them to learn from my mistake. I tell them that I am HIV positive because I never used condoms. I also advise them to go and get tested for HIV.”

*(Female PLWHA, 56)*

#### 4.2.6. Ownership and Responsibility

Instead of passively regarding it as the responsibility of their bridging partners, it is indispensable that the household feels responsible and confident that they have the strength to effectively respond to the challenges of living with HIV/AIDS. While some respondents see retention in care and treatment adherence as a responsibility shared between them and other household members, others see it as the sole responsibility of their household members. The male patients in the sample, in particular, saw it as the responsibility of their partner or mother. However, most of the respondents saw disease management as their own responsibility, while acknowledging the importance of support for their participation in the care continuum:

“It is my responsibility, but people in the house also remind me not to forget my clinic appointments.”

*(Female PLWHA, 42)*

#### 4.2.7. Social Space for Dialogue and Critical Thinking 

For patients in this sample, the emotional burden of the disease starts when they receive the results of their positive test, rather than when they first experience HIV-related symptoms. The impact of the positive test on the respondent is dependent on a number of factors, such as the expected consequences of the news for a patient’s sex life and social life. In this regard, key to the development of an HIV/AIDS competent household is not only disclosure of one’s HIV status, but also the need to go further in breaking the silence around HIV/AIDS and its implications. Indispensable to this is the creation of an atmosphere in which individuals feel comfortable to have an effective dialogue on the disease and its implications for the life of the patient and the household. This study found that a pre-existing culture of open communication facilitates such a climate in which the patient can talk with other household members in an informed way about the disease and its consequences at the individual and household levels. Another household member who lives openly and positively with HIV/AIDS also helps to create a social space in which the respondent feels comfortable to talk about the concerns accompanying his or her status:

“She [partner] knows everything because she went for counseling. They told her that anything can happen. If something goes wrong with me, she says ‘no man, don’t worry about those things’. You are aware that you might get it. Otherwise, those things I already knew and [if] I have a problem I ask her, she tells me about these things, she knows about them.”

*(Male PLWHA, 46)*

#### 4.2.8. Solidarity and Common Purpose 

Another aspect of an environment that is responsive to HIV/AIDS is a sense of solidarity and common purpose that allows household members to reach out to each other and tackle the impact on the household and the individual patient together. When the household builds a sense of solidarity and common purpose, this can provide additional support for the patient in their midst. For example, in our sample, household members assisted the patient with daily household tasks (e.g., by doing their laundry, washing them, or cooking for them), provided material support (e.g., financial or food), or helped in other ways (e.g., looking after children when the PLWHA went to the clinic). In addition, they also helped with more specific disease-management tasks, monitoring the treatment, reminding the patient about visits to the clinic, accompanying them to the clinic, fetching their medication, or helping them to accept their status and giving them emotional support—especially when others in the household were also openly positive, based on a shared experience:

“He [cousin] motivated me and said nothing will change, I am his family. He will support me right through.[cries]”

*(Female PLWHA, 27)*

Other household members can also stimulate adherence indirectly, as some respondents in the sample indicated they took ART as they did not want to die and consequently leave their parents or children behind. Some female patients in this study indicated that they adhered to their treatment because they wanted to protect their unborn child from becoming infected. Furthermore, the death of others in the social environment as a result of failing to adhere to their ART was for some patients a motivation to follow their treatment correctly. 

#### 4.2.9. Positive Living

As illustrated in Figure 1, recognition of the reality of HIV/AIDS and the creation of a trusting and safe environment to respond to this disease are important to build an HIV/AIDS competent context. Such an environment enables more effective HIV/AIDS management by mobilizing adequate care and support and by reducing other household members’ vulnerability to infection. In such an atmosphere, it is easier for respondents to deal with HIV/AIDS-related markers, such as the treatment adherence support visits of a CHW. The patients in the sample also felt more free to follow their ART at home, instead of deploying strategies to keep ART a secret:

“She [partner] understood and was not angry at all. All what she did was to encourage me to go and take treatment at the clinic. She gave me her support telling me that the HIV virus doesn’t kill people if they take treatment. But it happens to be the people who kill themselves by not taking treatment.”

*(Male PLWHA, 45)*

In an HIV/AIDS competent household, the members are more likely to feel confident to seek information about prevention or testing. Furthermore, an atmosphere of dialogue and critical thinking is vital to give individuals the voice to challenge aspects that place their health at risk, such as the need for condom use. The acknowledgment of the presence of HIV/AIDS in the household triggers preventive behavior by the respondents to their partner and children. Such behavior includes: choosing to use condoms systematically, not sharing needles and razor blades, reducing the number of concurrent sexual partners, informing health care workers when they have a wish to become pregnant, and by prevention of mother-to-child transmission (PMTCT). In this regard, the positive consequences of living in an AIDS competent household reach further than its impact on the PLWHA, as the health-enhancing practices of the PLWHA might also reduce the likelihood of a new HIV infection within the household:

“Before I got tested, I used to have sex without a condom. But now I use it regularly. After I discovered that I am HIV positive, I told myself that I should stick to one partner and I am not interested to be engaged into having sex.”

*(Male PLWHA, 22)*

Such an HIV/AIDS competent climate is not only important for health-enhancing, HIV-related behavior such as HIV-preventive behavior and accessing care services, but also to enhance the quality of life of the PLWHA and their household members. Various patients indicated that they and their household members lived a much healthier life after their positive diagnosis than before their treatment, for example, by limiting or stopping their use of alcohol.

#### 4.2.10. Dynamic 

Living in a household that is HIV/AIDS competent can stimulate other household members to be voluntarily counseled and tested. For example, some respondents disclosed their status within a climate of open communication with the aim of convincing their fellow household members to be tested. This feedback loop is an important step towards a sustainable response to HIV/AIDS-related challenges. Regular retesting is required so that each household member knows his or her HIV status. A newly identified positive patient in the household needs to disclose his or her status as well, which will be facilitated when living in an HIV/AIDS competent household, as individuals who feel supported by positive HIV-related precedents will focus more on the positive outcomes of disclosure and will be less likely to worry about the possible negative consequences. 

Indispensable to long-term success is that households are HIV/AIDS competent in a sustainable manner. To sustain their competence, households should also adapt to developments in the field of HIV/AIDS, such as the roll-out of the fixed-dose combination. Adaptations may need to be made in their own social context as well, such as when there is a new HIV infection in the household or when the PLWHA moves. In our sample, the PLWHA often came from the Eastern Cape, moving to the townships on the outskirts of Cape Town for economic reasons. Other respondents moved because they were offered care by that household when they became ill. In such a situation, a positive step towards HIV/AIDS competence in the household is taken:

“I moved here to stay here after when I was sick, because I have noticed that my son is the one who would do better to look after me.”

*(Female PLWHA, 56)*

When respondents start their life in a new environment, the process leading to HIV/AIDS competence in the new household has to start again. In this regard, the road to HIV/AIDS competence is influenced by the pre-existing knowledge of the patient and the positive or negative HIV-related precedents that the patient has already experienced. As one respondent testified:

“No they don’t know here, but in the Eastern Cape I was open about it because even in my neighborhood in Eastern Cape they come to me and ask me how I do it and I told them. [So] they go to clinic, they tested.”

*(Female PLWHA, 31)*

### 4.3. Barriers 

The development of HIV/AIDS competence in the household may be interrupted at any moment due to both HIV and non-HIV-related factors—both at the personal and the household level.

#### 4.3.1. Personal Barriers for PLWHA

There are several personal barriers to be faced by PLWHA on the road to HIV/AIDS competence in the household. Due to disbelief or because respondents moved from the Eastern Cape to Cape Town, several respondents had various HIV tests in different health facilities or during outreach testing in the community. Others, who reacted with disbelief or denial to their positive test results, put the care continuum on hold for several years. They ignored their positive status until they developed new symptoms, became pregnant or became so ill that it impacted on their daily functioning, leading them to return to the health facility for retesting. Except for one respondent, none of the patients disclosed their status when not believing the results—so the move towards HIV/AIDS competence in the household did not begin. While an open personality of the patient may have a facilitating effect, a closed personality can also make people more inclined to keep their status hidden. Furthermore, internal stigma deterred a respondent from disclosure. Acceptance of one’s positive status is a process that needs time. The more the patients in the study accepted their positive status, the more likely they were to tell others. Moreover, the absence of markers of HIV/AIDS fosters secrecy on the part of PLWHA. As some PLWHA only had themselves tested when they were already very ill, they had to start ART immediately after their positive test. This leaves patients little time to process the news and to disclose their status, even though they have ART medication in the house:

“The thing that makes it difficult with disclosure is that one goes to the clinic and gets counseling and understanding, but [he] doesn’t accept his own status. So if he can start by accepting, it will be easy to disclose to the family first, before outside.”

*(Community Health Worker, Health Facility 4)*

While it is important that household members develop a sense of ownership and responsibility to deal with the challenges of HIV/AIDS, it is indispensable that the patient also feels responsible for the self-management of his or her disease. Patients in the sample had ceased treatment in the past for periods of several months to more than a year, for various reasons, such as the presence of side effects, going on holiday to the Eastern Cape without sufficient medication or a transfer letter, moving to Cape Town, treatment fatigue, or drug or alcohol abuse. Most of them restarted ART when they became ill or pregnant. This can discourage household members from continuing their support:

“Some families are more supportive, especially when the patient is not drinking. Because when the patient is drinking, when she will be sober she will obey them. But on weekends, she will say ‘this is my life, you are not affected by this.’”

*(Community Health Worker, Health Facility 2)*

#### 4.3.2. Barriers at the Household Level

Several barriers along the road to HIV/AIDS competence can be identified within the household. First, when fearing stigmatization, the PLWHA will try to present him- or herself as HIV negative by adopting a hybrid identity. The household context plays an important role in this. Second, if a patient decides not to disclose to some or all household members, a burden of secrecy is created, which inhibits the development of HIV/AIDS competence. Third, if a patient decides to disclose, disbelief or a negative reaction can hamper the development of HIV/AIDS competence. Fourth, poverty can also challenge the development of HIV/AIDS competence in the household:

“The stigma starts in the home. The people who are supposed to support this person are the ones who discriminate against her/him, you know. So it’s a big problem.”

*(Community Health Worker, Health Facility 4)*

#### 4.3.3. Influence of a Negative Household Context on Disclosure

While for some patients the request for them to bring a treatment buddy to the counseling session forced them to disclose, others did not bring someone. Various reasons are given, such as lack of social support or unwillingness to disclose at that time. A household context characterized by negative HIV-related precedents, such as discrimination against another HIV-positive household member, inhibits disclosure and thus the development of HIV/AIDS competence. In a context characterized by lack of HIV-related knowledge, household members were less likely to take the first step on the road to HIV/AIDS competence themselves, as they were also not able to understand the visible markers of HIV/AIDS correctly, such as symptoms, treatment or the latter’s side effects. Lack of knowledge can also fuel stigma, as one community health worker describes:

“Some they have reasons [not to disclose]. They will say with their family quarrelling, they will tell other people that you are HIV positive. And some, if they are staying with people who don’t know HIV, they will put aside plates, spoons, all these utensils we you use they put aside so that they don’t want to share.”

*(Community Health Worker, Health Facility 2)*

In addition, negative non HIV-specific household dynamics, such as households characterized by physical abuse or a lack of emotional connectedness or trust, discourage patients from sharing their status. When there is no open communication within the household—especially in combination with alcohol abuse—the respondent’s control over their hybrid identity is threatened, which inhibits disclosure. Moreover, within such a discouraging context, the patient is not always able to keep control of his or her hybrid identity, since other household members may find out about the positive status through gossip, for example:

“We did ask why she doesn’t want the children to know. She just said ‘no they are aggressive and they are drinking too much’. She can’t tell them because they are going to swear at her. And she is scared they maybe going to abuse her.”

*(Community Health Worker, Health Facility 4)*

However, a good household climate does not guarantee disclosure. By not disclosing, some respondents felt that they could protect household members from emotional distress or external stigma, especially their grandparents. 

#### 4.3.4. Consequences of Non-Disclosure in the Household

Some patients who are in control of their hybrid identity do not disclose their status to their household but only to a person outside it, such as a friend, a neighbor or family elsewhere. When a patient does not disclose to some or all of the household members, a burden of secrecy is created. Non-disclosure may allow people to deny the reality of HIV-related illness and the need for behavior change. As a consequence of being unaware of the presence of the disease, the possibility of tapping into support from the household members is inhibited. The respondent’s own acceptance of their positive status is also challenged by the burden of secrecy, as this patient testifies:

“My family was so excited about my baby but I was stressing too much because I never disclosed my status to anyone at home. I was always crying and wanted to be alone at all times. You won’t believe it when I say that I woke up the other day; I went to the shop to buy poison that is meant to kill rats. I mixed the poison with water then I drank it. I collapsed and was admitted to Jooste Hospital. I was so stressed to find out that I am HIV positive.”

*(Female PLWHA, 36)*

Furthermore, non-disclosure to some or all household members forces the patient to conceal the visible markers of the disease, as these undermine hybrid identity management. This becomes particularly challenging when both partners are positive and unwilling to disclose to each other. We found that when some patients started ART, they developed strategies to conceal this HIV/AIDS marker. Some hid the medication in a bag of vitamins, others in a private room such as the bedroom, while others used TB as a disguise for their positive status. Respondents who did the latter, mentioned that by the time they had finished their TB treatment, their ART had also been effective in making them look healthy again, so they could keep living with HIV in secret. Lack of HIV-related knowledge in the household may assist a patient in this. However, for some patients, the only way they could preserve their hybrid identity was to stop treatment. Non-disclosure not only impacts on treatment but also on the prevention of transmission to others in the household. PLWHA must develop strategies to prevent transmission to others if they are concealing HIV/AIDS:

“I asked my boyfriend to use protection and he agreed because I lied to him and said that I have a problem in my womb. I take my medication in front of him but he doesn’t know what that medication is for. He only knows that I collect medication for high blood pressure and I have a problem in my womb.”

*(Female PLWHA, 27)*

#### 4.3.5. Disbelief or a Negative Response to Disclosure

In this study it was not only PLWHA who reacted with disbelief to their positive test, as some household members also reacted in a similar manner when the patient disclosed his or her status to them. Disbelief, fuelled by a lack of knowledge or understanding about the illness and misconceptions about HIV transmission routes in the household, can foster HIV/AIDS stigma and inhibit preventive measures, such as the use of condoms. When household members in denial were confronted with markers of the disease, such as symptoms associated with a low CD4 cell count or the ART medication, they started to believe the reality of HIV/AIDS in their household:

“He [partner] did not believe it because I never lost weight. (…) He only believed it last year when he saw that I continued with my treatment.”

*(Female PLWHA, 38)*

As disclosure is based on the careful weighing of advantages and disadvantages, adopting a hybrid identity protected most patients in this sample from negative responses to disclosure. However, some household members did respond negatively, such as blaming the PLWHA for bringing HIV/AIDS into the house, and making negative judgments and spreading of rumors about the patient outside the household. The first household member to be identified as HIV positive is more often labeled as the person who is responsible for bringing the disease into the house. As women are more frequently identified as HIV positive before male household members, it was the female respondents who more often experienced such accusations and blame on disclosure. However, while some patients in this sample wondered about the source of infection, others blamed their partner. A negative response can negatively impact on the patient’s ability to self-manage their disease and adhere to treatment:

“I was under a lot of stress because there were people who were judging me, so I stopped [ART] […] They were judging me by insulting me about my HIV status. And that I am going to infect my husband, because my sisters-in-law were saying that.”

*(Female PLWHA, 27)*

#### 4.3.6. Poverty

In our sample, poverty often ruled over health. If a patient had a job, this job took priority over ART. For example, the patient might miss a clinic appointment to collect medication if they were working. When living in an HIV/AIDS competent household, PLWHA are more likely to receive support in combining work and ART as other household members could pick up the medication. However, even in an HIV/AIDS competent household, positive living can be challenged by resource constraints: in some instances, patients did not want to take their ART on an empty stomach but had no food in the house. While some patients lost their job as a result of being ill, for others the positive test meant a new income in the household through a disability grant. Patients and CHWs in this study testified that some PLWHA stop their treatment to let their CD4 cell count drop so that they can access a disability grant:

“They wait for their medication, but others they don’t because they say that they want to go and drink alcohol so that their CD4 count drops so that they can be able to apply for the grant.”

*(Female PLWHA, 22 years)*

### 4.4. Not a Panacea

Despite the various barriers, the results show that the household can be an important health-enabling environment. However, one patient in the study did not have other household members. He received most support from his sister, who was living in a township nearby. Moreover, a less HIV/AIDS competent household does not necessarily result in bad self-management by the PLWHA, nor is an HIV/AIDS competent household a guarantee of success. For example, one respondent who was forgetful was reminded so often by household members that she took more pills than prescribed; other patients felt that the support they received was too patronizing; while other patients perceived support as stigmatizing, as it was a marker and a reminder of their disease:

“Sometimes when you are HIV, you lose hope, you don’t have much respect for yourself, even for others that are around you. Because the people you hurt most are those who are close to you, that care for you. You don’t want people to treat you as if like, you know, you are sick.”

*(Male PLWHA, 30)*

## 5. Discussion 

Although important steps have been taken in the response to the pandemic, persistent and emergent challenges remain in the still unfolding history of HIV and AIDS. Despite the remarkable progress in the fight against the disease, a decrease in the number of new HIV infections and AIDS-related deaths, as well as an increase in access to antiretroviral therapy [54,55], 2.1 million people became newly infected with HIV in 2013 and 1.5 million people died from AIDS-related causes worldwide in the same year [56]. To respond to these challenges, sufficient attention should be paid to chronic disease care in order to support patients throughout the care continuum [7,8,9]. To provide chronic disease care within the context of human resource shortages, there is a need to draw on the patient’s social environment to build a health-enabling context that fosters health in the long term [57]. This need to increase HIV/AIDS knowledge, reduce stigma, encourage HIV testing, improve health care seeking behavior, and stimulate safe sexual practices in the social environment is described in the literature as the need for *AIDS competence*. On the basis of a socio-ecological perspective, we have argued that we should not only focus on the dominant community-level approach but also on the household level when building comprehensive social contexts that enable and support the choice of health-enhancing practices. The aim of this article was to conceptualize such an HIV/AIDS competent household.

The article shows that the household has the potential to form a health-enabling environment in which the patient can be supported across the care continuum in a sustainable manner. However, the road to HIV/AIDS competence is fragile and prone to barriers at different levels. In addition to positive HIV-related precedents and HIV-related knowledge, positive pre-existing household dynamics helpfully influence the development of HIV/AIDS competence in the household. In such a context in which they have less fear of stigmatization, PLWHA are more likely to embrace the identity of an HIV patient on ART, rather than present themselves as HIV negative [13]. By adopting a hybrid identity, a PLWHA will try to act as a gatekeeper who regulates the involvement of the household in his or her HIV/AIDS disease management, depending on their own perception of the environment. In the majority of the households in this sample, the PLWHA was the change agent who created awareness and openness about the disease and the need for behavior change. Our results indicate that women are more likely to be the drivers behind the move towards HIV/AIDS competence. Pregnancy or family planning led women in the sample to health facilities, where testing was initiated by health care providers. In this study, such provider-initiated testing meant women were often tested earlier than their male partners. These findings are in line with data from the 2010–2011 South African national HIV counseling and testing campaign, which showed that men represented only 30% of those tested [58]. Despite their fear of accusations and blame, especially when economically dependent, women who decided to disclose were found to be more likely to take on the role of health advisors to their partners or children than the men included in this study. These results add to the existing literature on the gendered nature of care [25,41,59].

Five resources identified by Campbell *et al.* [19], whose presence or absence serve to facilitate or hinder AIDS competence in the community, can also be found at the household level. Awareness of HIV/AIDS through disclosure and the acknowledgment of the disease in the home by supportive household members are prerequisites for the construction of an HIV/AIDS competent household. When there is an awareness of the HIV infection, household members can share the knowledge learned from bridging social capital—in line with the disclosure process model of Chaudoir *et al*., who state “individual disclosures can also affect the broader social context in which disclosers live” ([60], p. 1625). Bridging social capital is required not only for accurate information but also to access resources from outside that can support households in their effort to support the patient. However, rather than passively regarding this as the responsibility of these bridging partners, a sense of responsibility about HIV/AIDS and confidence in the household’s strengths is indispensable. Key to the development of an HIV/AIDS competent household is to go further than mere disclosure in breaking the silence around HIV/AIDS. This requires a context of solidarity and common purpose, which will allow household members to further build a context in which more effective HIV/AIDS management is possible, making prevention and treatment part of the daily life of the household. A feedback loop might also be created, in which other household members are motivated to seek counseling and be tested, as well as disclose their status, which is an important step towards a sustainable response to HIV/AIDS-related challenges. Such HIV testing is “the critical, cost-effective first step in the cascade of HIV treatment, as well as the gateway to other prevention and care interventions, such as male circumcision, prevention of mother-to-child HIV transmission, and prophylaxis of opportunistic infection” ([6], p. 60).

While the literature on AIDS competent communities has been an important inspiration for our conceptualization of HIV/AIDS competent households, two differences can be noted. First, as the term suggests, in the conceptualization of AIDS competent communities only *AIDS* is emphasized. We decided to use the phrase HIV/AIDS competence rather than AIDS competence, in line with Mathiot’s remark on the Self-Assessment Framework for AIDS Competence [31]. Due to access to antiretroviral treatment, PLWHA who follow the care continuum now face a life with HIV/AIDS as a chronic medical condition, rather than an acute, fatal disease that reaches the AIDS stage [61,62]. Second, in contrast to an AIDS competent community, disclosure by the PLWHA is pivotal for the development of HIV/AIDS competence in the household. While disclosure is not a necessary condition to reap the benefits of an HIV/AIDS competent community, it is a condition *sine qua non* for an HIV/AIDS competent household. Disclosure opens the gate to the road leading to HIV/AIDS competence. The PLWHA plays a key role in this, being the change agent who starts the move towards HIV/AIDS competence by bringing awareness of the disease to the household. 

There is a reciprocal relationship between AIDS competent communities and the households that are part of such a community. To begin with, an AIDS competent community provides an important facilitating context for households to gain more HIV/AIDS competence, providing opportunities to access bridging social capital, which in turn results in access to testing, information, prevention methods and treatment, among other things. Lamboray and Skevington illustrate this with the example of household members discussing HIV/AIDS “as a result of their children bringing in new information and ideas from school, from participation in community meetings and from awareness-raising entertainment that occurs periodically in adult gatherings” ([10], p. 518). An AIDS competent community also plays an important role in gradually normalizing and demystifying HIV/AIDS. In our study, the normalization of the disease due to its high prevalence in the community often assisted the respondents and the other members of their household to accept the reality of their disease and its treatment. However, a community in which an HIV/AIDS competent household lives, can also be disadvantaged by stigma, poverty, poor infrastructure and limited access to basic services. Fuelled by stigma, some patients avoided health facilities located in their own community. At the same time, however, households can also contribute to the development of AIDS competence in the community. Members of an HIV/AIDS competent household are more confident about making their story public to their surrounding community. They attempt to encourage others to voluntarily have themselves tested, to practice safe sex and to adhere to the treatment regime. By testifying about their own lived experiences to others, they can have a positive impact on the community, in terms of its understanding and acceptance of HIV and AIDS. 

It is important to note the limitations of this study. First, no household members were asked to participate in the study because we wished to safeguard the confidentiality of the patient. However, some household members joined in the interview spontaneously—which may be an indication of the openness to HIV/AIDS of that particular household. It would be interesting to include the perspective of household members of PLWHA in future research, while avoiding unnecessary disclosure of the patient’s status. Second, a selection bias has to be acknowledged in this study. All of the respondents who were willing to participate in the interview were on ART—except one, who had ceased treatment before the start of the study—and were receiving additional treatment adherence support from a CHW. The study was unable to survey patients who avoided treatment adherence support, for instance by not accepting visits during counseling or by providing the wrong address, who were not present at the participatory observation visit or who cancelled the interview. These PLWHA are likely to be the ones who are most difficult to reach in the treatment adherence support program, while perhaps also being those who need the support the most. Despite the fact that the care continuum of a number of respondents had been interrupted in the past, these findings cannot be generalized. In this regard, it has to be noted that these results support Hallet and Eaton’s modification of the traditional linear care continuum, allowing for multiple paths through the stages of the HIV care continuum [4]. By focusing on patients at different stages of the care continuum and in different settings, future research could make interesting progress on the conceptualization of HIV/AIDS competent households. Moreover, valuable insights could be gained by following households for an extended period of time from diagnosis onwards. Longitudinal research could advance our understanding of how HIV/AIDS competent households develop over time and under which conditions, as household boundaries and composition are unlikely to remain stable [39,41,63,64,65,66,67]. More specifically, the relationship between migration and care needs at household level should also be investigated in greater depth. Our results appear to support Niehof’s statement that “care needs also initiate changes in living arrangements, household means and the division of household labor, as are evident in households affected by AIDS” ([38], p. 495). Our results imply that female PLWHA are important change agents in households, but additional research focusing on the gender aspect of HIV/AIDS competent households would provide further input on this notion. As some patients rely on people outside the household (e.g., extended family elsewhere, friends or neighbors), further research is also required to determine why these patients seek support from these relationships. This knowledge may add to the further refinement of our conceptualization of the HIV/AIDS competent household. In addition, an HIV/AIDS competent household can also be an important source of support for children and adolescents living with HIV/AIDS. Further research should explore this topic from their perspective. Future research should also focus more on the role of household economics in the building of HIV/AIDS competence. For a sustainable response to the HIV/AIDS epidemic, it would also be interesting to investigate how HIV/AIDS competence develops in households without the presence of an HIV-positive person.

From a theoretical point of view, this article introduces the intermediate household level into AIDS competence research, in accordance with socio-ecological theory. To the best of our knowledge, this study is the first to conceptualize an HIV/AIDS competent household. To date, very few studies have assessed the impact of household dynamics on the care continuum [68,69,70,71]. This article shows that social-scientific research should also incorporate the intermediate role of households in constructing a health-enabling environment which helps patients to successfully navigate the care continuum and thus take maximum advantage of the opportunities created by ART scale-up, as articulated by Wouters [13]. The article highlights the fact that households are capable of managing disease even in times of transition or adversity in the face of the socio-economic impacts of HIV/AIDS on their daily lives [64,72]. Furthermore, this article adds to existing research on the importance of the social context in HIV care [20,25,63,73,74,75] and to the limited research carried out to date in countries with high HIV prevalence, such as those in Sub-Saharan Africa [25,38,41,76], where such studies should have high research priority. Unlike the majority of previous research on disease management, this study conceptualized an HIV/AIDS competent household within the framework of multiple interwoven factors [57].

From the perspective of practice and policy, the article addressed the need for “pre-intervention research” on household factors that affect disease management by PLWHA ([12], p. 26). These results raise the significant point that the formal care continuum is almost completely isolated from the household. The study illustrates that more attention should be paid to guiding the patient in the process of involving their household in the care continuum, in order to optimally capitalize on the strengths of households. In this regard, attention should be focused on mobilizing the patient’s natural support system to improve disease management—as well as the health and well-being of patients and all household members—by enhancing emotional connectedness and increasing mutually supportive interactions among household members. The results show that policy programs are also needed to support the household to overcome the various barriers on the road to HIV/AIDS competence. Support is needed to reduce the household’s potential negative effects on a PLWHA within the care continuum, such as helping to minimize intra-household stigmatization. Policy programs should be developed to support the household to manage “the continuing stresses inherent in chronic disease management as a team, rather than as individuals” ([12], p. 25).

## 6. Conclusions

The roll-out of ART has redefined HIV/AIDS as a chronic disease rather than a terminal illness. Living with a chronic condition is “complex and requires integration of self-management behaviors into the lifestyles of individuals and household” ([33], p. 218). Despite the fact that the road to HIV/AIDS competence is fragile and prone to various barriers, this article shows that the household has the potential to be a health-enabling environment which provides sustainable support to the patient on his or her care continuum. 
